# Integrated analysis of ceRNA network and tumor-infiltrating immune cells in esophageal cancer

**DOI:** 10.1042/BSR20203804

**Published:** 2021-05-27

**Authors:** Yuhua Chen, Hao Zhou, Zhendong Wang, Zhanghao Huang, Jinjie Wang, Miaosen Zheng, Xuejun Ni, Lei Liu

**Affiliations:** 1 Nantong Health College of Jiangsu Province, Nantong 226010, Jiangsu, China; 2 School of Medicine, Nantong University, Nantong 226001, Jiangsu, China; 3 Department of Medical Ultrasound and Radiology, Affiliated Hospital of Nantong University, Nantong 226001, Jiangsu, China; 4 Department of Pathology, Affiliated Hospital of Nantong University, Nantong 226001, Jiangsu, China

**Keywords:** ceRNA network, Esophageal cancer, Immune infiltration, Prognostic model, TCGA

## Abstract

Background: Esophageal cancer (ESCA) is one of the most commonly diagnosed cancers in the world. Tumor immune microenvironment is closely related to tumor prognosis. The present study aimed at analyzing the competing endogenous RNA (ceRNA) network and tumor-infiltrating immune cells in ESCA.

Methods: The expression profiles of mRNAs, lncRNAs, and miRNAs were downloaded from the Cancer Genome Atlas database. A ceRNA network was established based on the differentially expressed RNAs by Cytoscape. CIBERSORT was applied to estimate the proportion of immune cells in ESCA. Prognosis-associated genes and immune cells were applied to establish prognostic models basing on Lasso and multivariate Cox analyses. The survival curves were constructed with Kaplan–Meier method. The predictive efficacy of the prognostic models was evaluated by the receiver operating characteristic (ROC) curves.

Results: The differentially expressed mRNAs, lncRNAs, and miRNAs were identified. We constructed the ceRNA network including 23 lncRNAs, 19 miRNAs, and 147 mRNAs. Five key molecules (HMGB3, HOXC8, HSPA1B, KLHL15, and RUNX3) were identified from the ceRNA network and five significant immune cells (plasma cells, T cells follicular helper, monocytes, dendritic cells activated, and neutrophils) were selected via CIBERSORT. The ROC curves based on key genes and significant immune cells all showed good sensitivity (AUC of 3-year survival: 0.739, AUC of 5-year survival: 0.899, AUC of 3-year survival: 0.824, AUC of 5-year survival: 0.876). There was certain correlation between five immune cells and five key molecules.

Conclusion: The present study provides an effective bioinformatics basis for exploring the potential biomarkers of ESCA and predicting its prognosis.

## Introduction

Esophageal cancer (ESCA) is one of the leading triggers for cancer-related death in the world, and its incidence rate continues to rise [1]. The 5-year survival rate of ESCA is less than 15%, so it has poor prognosis and high mortality [2]. Due to the lack of effective early diagnosis, ESCA usually appears in the advanced stage. Patients can no longer swallow solid foods, and the clinical prognosis is poor [3]. Accurately assessing the prognosis of patients can provide them with personalized treatment and reduce the mortality of patients [4]. Therefore, further research is necessary to discover potential biomarkers to improve the diagnosis and prognosis.

Nowadays, immunotherapy and targeted treatment are widely used, which have become a significant way to improve the prognosis of ESCA patients [5]. The tumor microenvironment (TME) plays an important role in the formation, development, and treatment of tumors [6]. Immune cells are the major component of TME and closely associated with tumor occurrence. In addition, it has been proved that the prognosis and malignant degree of tumors are associated with the TME immune cells to a certain extent. The competing endogenous RNA (ceRNA) network was composed of mRNAs, lncRNAs, and miRNAs. The complex crosstalk of the ceRNA network has been verified in numerous diseases including cancer [7]. For example, it is reported that down-regulated lncRNA UCA1 acted as ceRNA to adsorb microRNA-498 to suppress proliferation and invasion of esophageal cancer cells by inhibiting ZEB2 expression [8]. Moreover, the crosstalk between the tumor cells and tumor-infiltrating immune cells is usually regulated via ceRNA networks [9].

In the present study, we obtained the expression profiles of mRNAs, lncRNAs, and miRNAs from the Cancer Genome Atlas (TCGA) database and established a ceRNA network on the basis of differentially expressed genes (DEGs) between tumor and normal samples. The fraction of different immune cells in ESCA was estimated by CIBERSORT. Then, we constructed two prognostic models according to the results of the ceRNA network and CBERSORT analysis. We further evaluated the relationship between the immune cells and the hub genes involved in the ceRNA network to find potential molecular pathways for the immunotherapy of ESCA patients.

## Materials and methods

### Data source

Gene expression profiles and the related clinical data of TCGA-ESCA patients were downloaded from the TCGA database (data release 23.0- April 7, 2020), including mRNA, lncRNA, and miRNA. We downloaded the FPKM value of gene expression of 160 ESCA samples and 11 normal samples. After excluding the samples that had missing clinical information, 159 samples with complete clinical information were finally obtained. Data acquisition was in accordance with TCGA publication guidelines.

### Differential gene expression analysis and construction of the ceRNA network

Using the edgeR package in R software, we identified differentially expressed mRNA (DEmRNA), miRNA (DEmiRNA), and lncRNA (DElncRNA) [10]. Genes with |logFC| > 1 and *P*.adjust < 0.05 were regarded as DEGs. MiRcode database was used to predict lncRNA–miRNA interactions [11]. Three databases, including miRTarBase, TargetScan, and miRDB, were performed to find the potential mRNAs for DEmiRNA [12–14]. Only the genes included in all three datasets were selected for further analysis. By crossing the predicted mRNAs with DEmRNAs, we obtained the mRNAs in the network. According to the results above, a ceRNA network was constructed and further visualized by Cytoscape [15].

### Functional and pathway enrichment analyses

To explore potential biological processes related to the DEmRNAs involved in the ceRNA network, we performed gene ontology (GO) and Kyoto Encyclopedia of Genes and Genomes (KEGG) enrichment analysis using the clusterProfiler R package [16]. The *P*.adjust < 0.05 displays statistical significance. The most significant GO terms and pathways were selected for visualization.

### Construction of a five-gene prognostic model based on the ceRNA network

The DEGs correlated with overall survival (OS) were screened via univariate Cox regression analysis with the survival R package. Then, lasso regression was employed to reduce dimensionality, and the most robust markers were selected to construct the prognostic model. The 10-fold cross validation was applied to define the optimal value of the penalty parameter λ [17]. Each patient’s risk score was finally calculated by multiplying the expression level of each gene by its Cox regression coefficient and adding these values.

### Immune infiltrate analysis

CIBERSORT is a calculation method for characterizing cell subsets of interest in high-dimensional genomic data derived from large tissue samples [18]. To explore the difference of infiltrating immune cells, we used CIBERSORT to evaluate the fraction of 22 kinds of immune cells in the ESCA samples and normal samples with a deconvolution algorithm [19]. Only samples with CIBERSORT *P*<0.05 could be used for further analysis.

### Construction of a prognostic model based on immune cells

We used univariate Cox regression analysis to determine the prognostic value of the 22 immune cells. Then, the key members of the immune cells were identified via Lasso and multivariate Cox analyses. The risk score of each patient was finally calculated via multiplying the abundance of each immune cell by its Cox regression coefficient and adding these values.

### The Co-expression and TIMER analysis

Pearson correlation analysis was performed to analyze the co-expression patterns between the immune cells and the key molecules involved in the ceRNA network. As a public resource, TIMER uses a deconvolution method to infer the abundance of immune infiltration from gene expression profiles [20]. Based on the TIMER website, we evaluated the correlation between the key molecules involved in the ceRNA network and six TME infiltrating cells including CD4+ T cells, dendritic cells, B cells, CD8+ T cells, neutrophils, and macrophages.

### Statistical analysis

Kaplan–Meier method was used to construct survival curves. The receiver operating characteristic (ROC) curve was plotted with the SurvivalROC R package. Statistical *P*<0.05 was considered statistically significant. R software (version 3.6.3) was used to perform all data analyses.

## Results

### Identification of DEGs in ESCA

**Figure 1:**
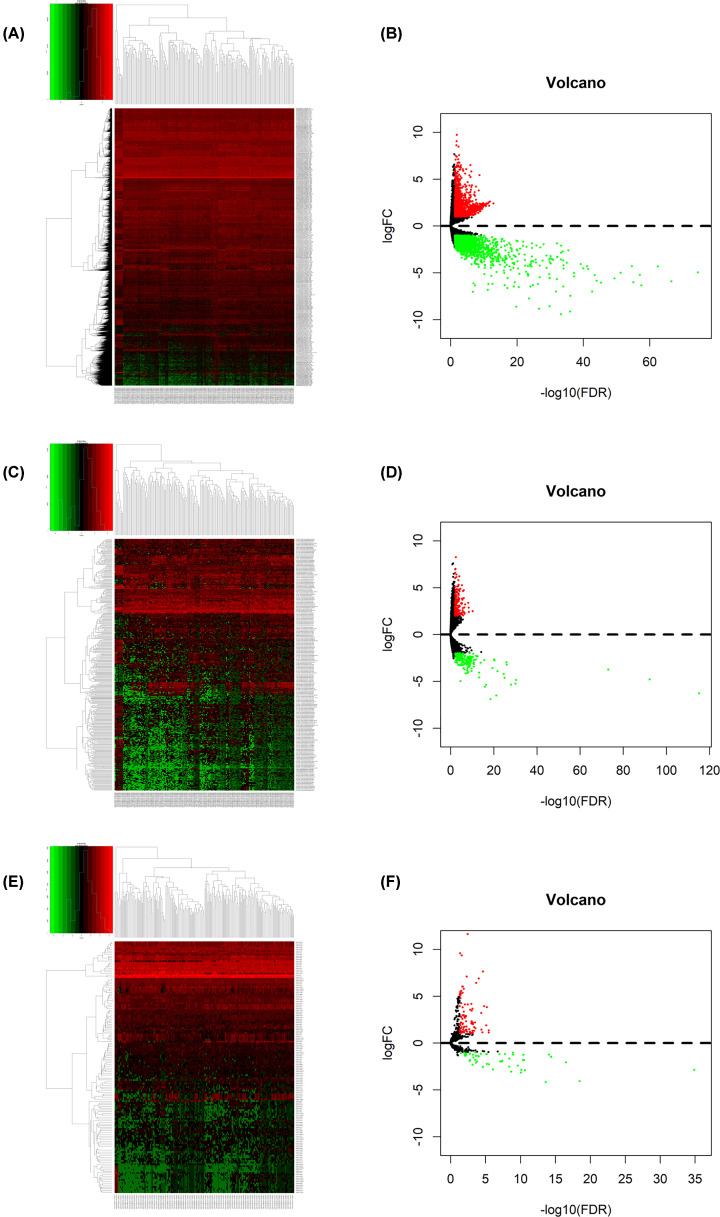
Differentially expressed genes between ESCA samples and normal samples (**A**) Heatmap and (**B**) volcano plot of differentially expressed mRNAs between normal and tumor samples. (**C**) Heatmap and (**D**) volcano plot of differentially expressed lncRNAs between normal and tumor samples. (**E**) Heatmap and (**F**) volcano plot of differentially expressed miRNAs between normal and tumor samples. Red points represent up-regulated genes. Green points represent down-regulated genes. Black points represent genes with no significant difference; ESCA, esophageal cancer.

A total of 3211 DEmRNAs, 381 DElncRNAs, and 144 DEmiRNAs were identified using the edgeR package in R software. Among them, 1461 lncRNAs, 19 miRNAs, and 1511 mRNAs were up-regulated and 554 lncRNAs, 28 miRNAs, and 803 mRNAs were down-regulated. The cut-off criteria was |logFC| >1 and *P*.adjust<0.05. Figure 1A–F showed the heatmaps and volcano plots.

### The ceRNA network building and functional enrichment analysis

**Figure 2:**
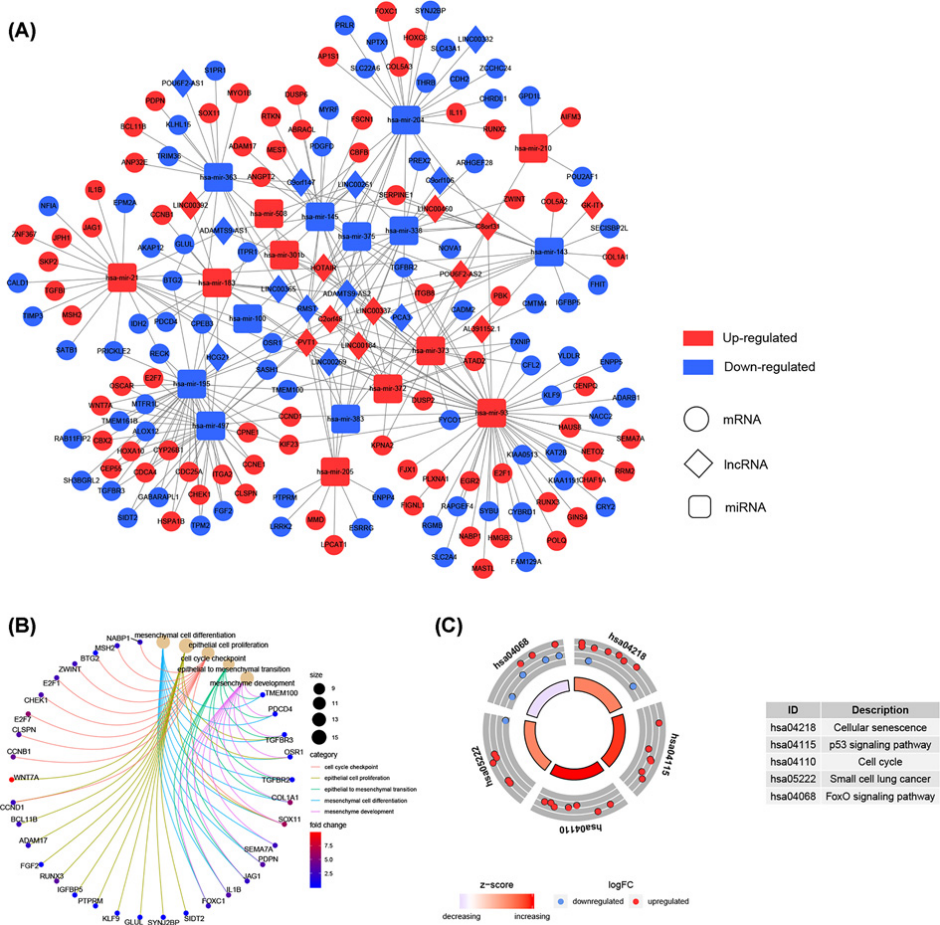
The ceRNA network and functional annotation (**A**) The ceRNA network of differentially expressed mRNAs, differentially expressed miRNAs, and differentially expressed lncRNAs; Red, up-regulated; Blue, down-regulated. Circle represents mRNA; Diamond represents lncRNA; Rectangle represents miRNA. (**B**) GO enrichment analyses of the mRNAs involved in the ceRNA network. (**C**) KEGG pathway enrichment analyses of the mRNAs involved in the ceRNA network; GO, Gene Ontology; KEGG, Kyoto Encyclopedia of Genes and Genomes.

With miRcode database, we predicted 115 pairs of interacting lncRNA–miRNA. Then, 1043 potential mRNAs included in all three datasets (miRTarBase, TargetScan, and miRDB) were predicted for DEmiRNAs. Finally, 147 target mRNAs were gained by intersecting the predicted mRNAs with DEmRNAs. To investigate how lncRNAs mediate mRNA by binding to miRNA in ESCA, we constructed the ceRNA network according to the results above, including 23 lncRNA nodes, 19 miRNA nodes, and 147 mRNA nodes (Figure 2A).

To gain further insight into the biological functions and potential pathways bound up with the ceRNA network, GO and KEGG pathway analyses were performed by clusterProfiler R package. The biological processes of GO term enrichment demonstrated that the DEmRNAs involved in the ceRNA network were particularly enriched in mesenchymal cell differentiation, epithelial cell proliferation, and cell cycle checkpoint (Figure 2B and Supplementary Table S1). In addition, the results of KEGG analysis suggested that the DEG-associated pathways were cellular senescence, p53 signaling pathway, and cell cycle (Figure 2C and Supplementary Table S2).

### Construction of a five-gene prognostic model based on the ceRNA network

**Figure 3:**
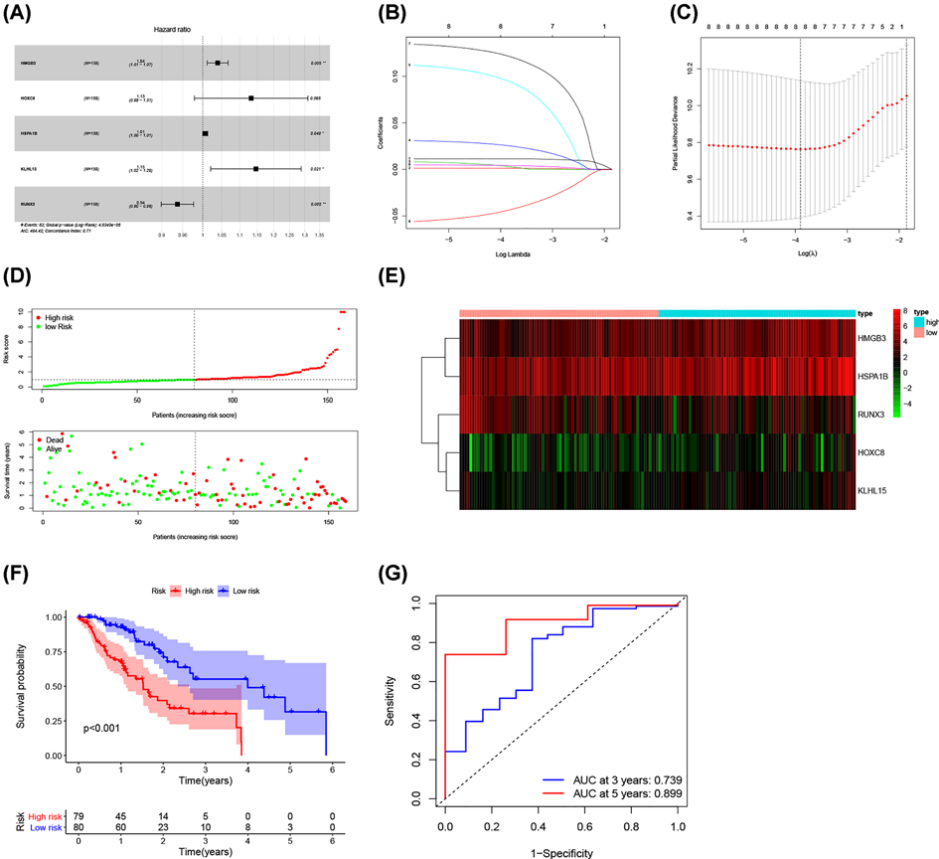
Construction of a five-gene prognostic model (**A**) The results of the multivariate Cox regression. (**B**) Least absolute shrinkage and selection operator (LASSO) coefficient profiles of the five key molecules. (**C**) Tuning parameter selection by 10-fold cross-validation in the LASSO model. The partial likelihood deviance was plotted against log(Lambda/λ), and λ was the tuning Parameter. (**D**) Risk score distribution and survival status. The black dotted line is the optimum cut-off dividing patients into low-risk and high-risk groups. The red curve represents high risk and the green curve represents low risk. The dots indicate the survival status, the red dot indicates the death of the patient, and the green dot indicates alive. (**E**) Expression heat map. (**F**) Survival curve for the low-risk and high-risk groups. (**G**) ROC curve analysis in the TCGA-ESCA cohort; ESCA, esophageal cancer; ROC, receiver operating characteristic; TCGA, The Cancer Genome Atlas.

Univariate Cox regression analysis was applied to screen the prognostic-related DEmRNAs involved in the ceRNA network. The results demonstrated that 8 of the 147 DEmRNAs were significantly correlated with OS. Then, Lasso Cox regression was used to identify hub survival-associated genes by reducing the dimensionality (Figure 3B,C). Finally, multivariate Cox regression analysis was performed to calculate the relative coefficients of the genes with the best prognostic value. The result indicated that HMGB3, HSPA1B, KLHL15, and RUNX3 were independent prognostic factors (*P*<0.05) (Figure 3A). Supplementary Table S3 listed the coefficients for each gene. The prognostic risk score for each patient was calculated. Then, patients were separated into high and low-risk groups basing on the median risk score. The survival analysis showed worse prognosis for patients in high-risk group (Figure 3F). ROC curve was performed to determine the predictive accuracy of this prognostic signature, the AUC value was 0.739 for 3-year survival and 0.899 for 5-year survival (Figure 3G). This revealed that this prognostic signature had good prediction accuracy for ESCA patients. The distribution of survival state showed that the number of deaths increased with the increase of risk score (Figure 3D). Heatmap showed the trend of gene expression in high- and low-risk groups (Figure 3E).

### Composition of immune cells in ESCA

**Figure 4:**
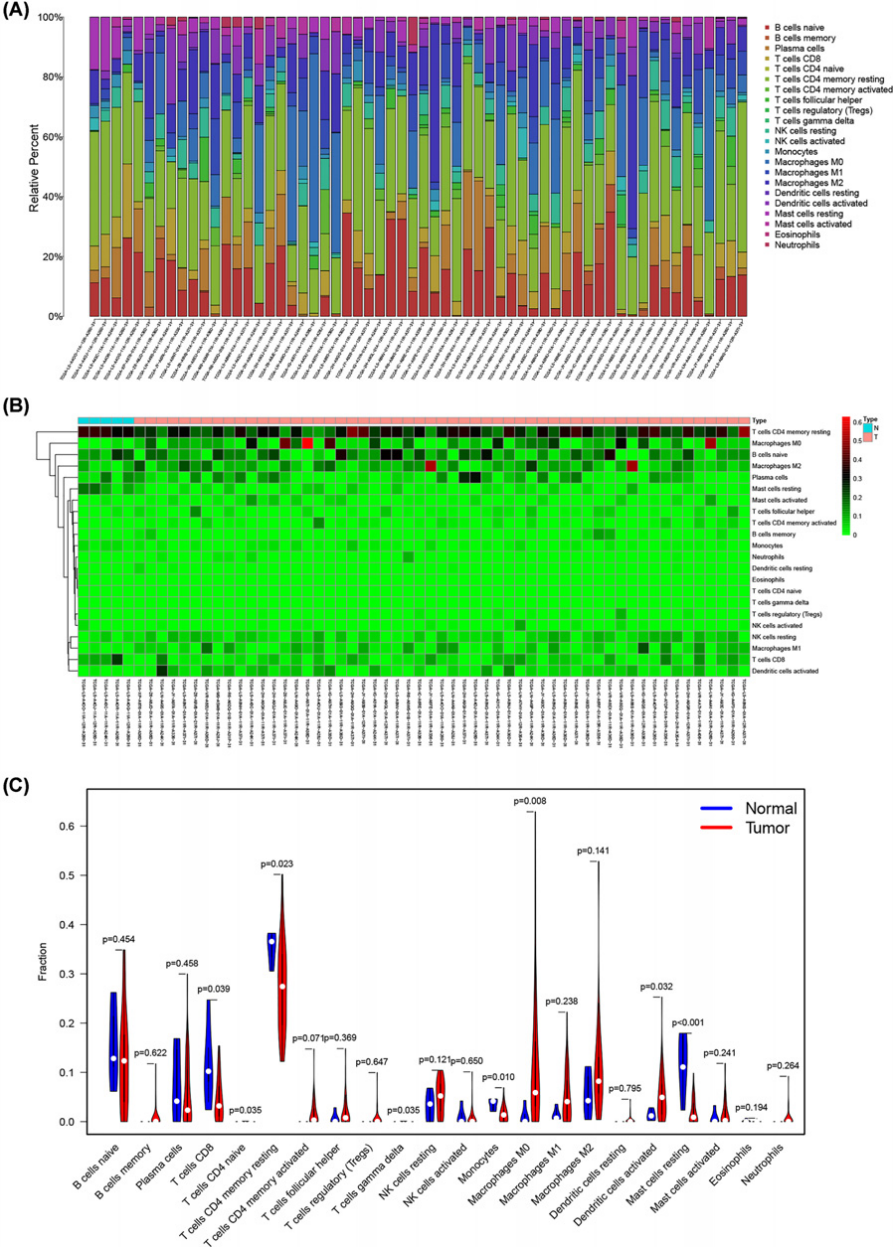
Evaluation of proportions of tumor-infiltrating immune cells based on CIBERSORT The (**A**) composition and (**B**) heat map of 22 subtypes of immune cells in ESCA. *X* axis represents ESCA samples. *Y* axis represents the proportion and abundance of immune cells. (**C**) Differences of 22 tumor-infiltrating immune cells between normal and tumor groups; ESCA, esophageal cancer.

The abundance of 22 kinds of immune cells was estimated to explore the difference of tumor microenvironment between ESCA samples and normal samples (Supplementary Table S5). Figure 4A,B showed the ratio of 22 immune cells detected via the CIBERSORT algorithm. The violin plot was performed by the Wilcoxon test. The results suggested that the infiltration levels of macrophages M0 (*P*=0.008) and dendritic cells activated (*P*=0.032) were obviously higher in ESCA samples, while CD8 T cells (*P*=0.039), CD4 T cells memory resting (*P*=0.023), monocytes (*P*=0.01), and mast cells resting (*P*<0.001) were significantly lower (Figure 4C).

### Construction of a prognostic model on the base of immune cells

**Figure 5:**
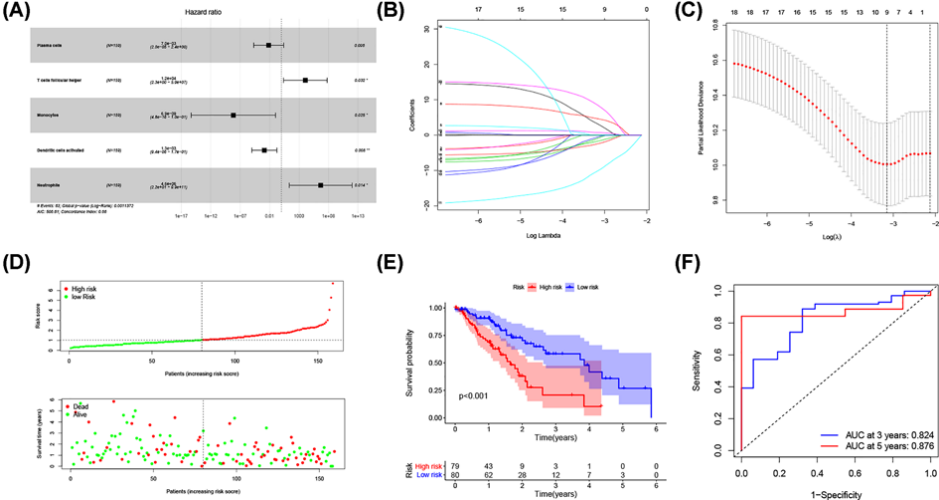
Construction of a prognostic model based on the immune cells (**A**) The results of the multivariate Cox regression. (**B**) Least absolute shrinkage and selection operator (LASSO) coefficient profiles of the five immune cells. (**C**) Tuning parameter selection by 10-fold cross-validation in the LASSO model. The partial likelihood deviance was plotted against log(Lambda/λ), and λ was the tuning Parameter. (**D**) Risk score distribution and survival status. The black dotted line is the optimum cut-off dividing patients into low-risk and high-risk groups. The red curve represents high risk and the green curve represents low risk. The dots indicate the survival status, the red dot indicates the death of the patient, and the green dot indicates alive. (**E**) Survival curve and (**F**) ROC curve analyses; ROC, receiver operating characteristic.

Univariate Cox regression analysis was used to screen the immune cells with prognostic value. Five immune cells including Plasma cells, T cells follicular helper, monocytes, dendritic cells activated, and neutrophils were identified by Lasso and multivariate Cox analyses (Figure 5A–C). A new prognostic signature was built basing on the five immune cells. The relative coefficients were calculated by multivariate Cox regression analysis and listed in Supplementary Table S4. Then, we calculated the patient’s risk score and separated them into high- and low-risk groups according to the median risk score. The survival analysis showed worse prognosis for patients in high-risk group (Figure 4E). ROC curve indicated good prediction accuracy with the AUC of 0.824 for 3-year survival and 0.876 for 5-year survival (Figure 4F). The survival status distribution graph indicated that the number of deaths increased with the increase of risk score (Figure 5D).

### The Co-expression analysis

**Figure 6:**
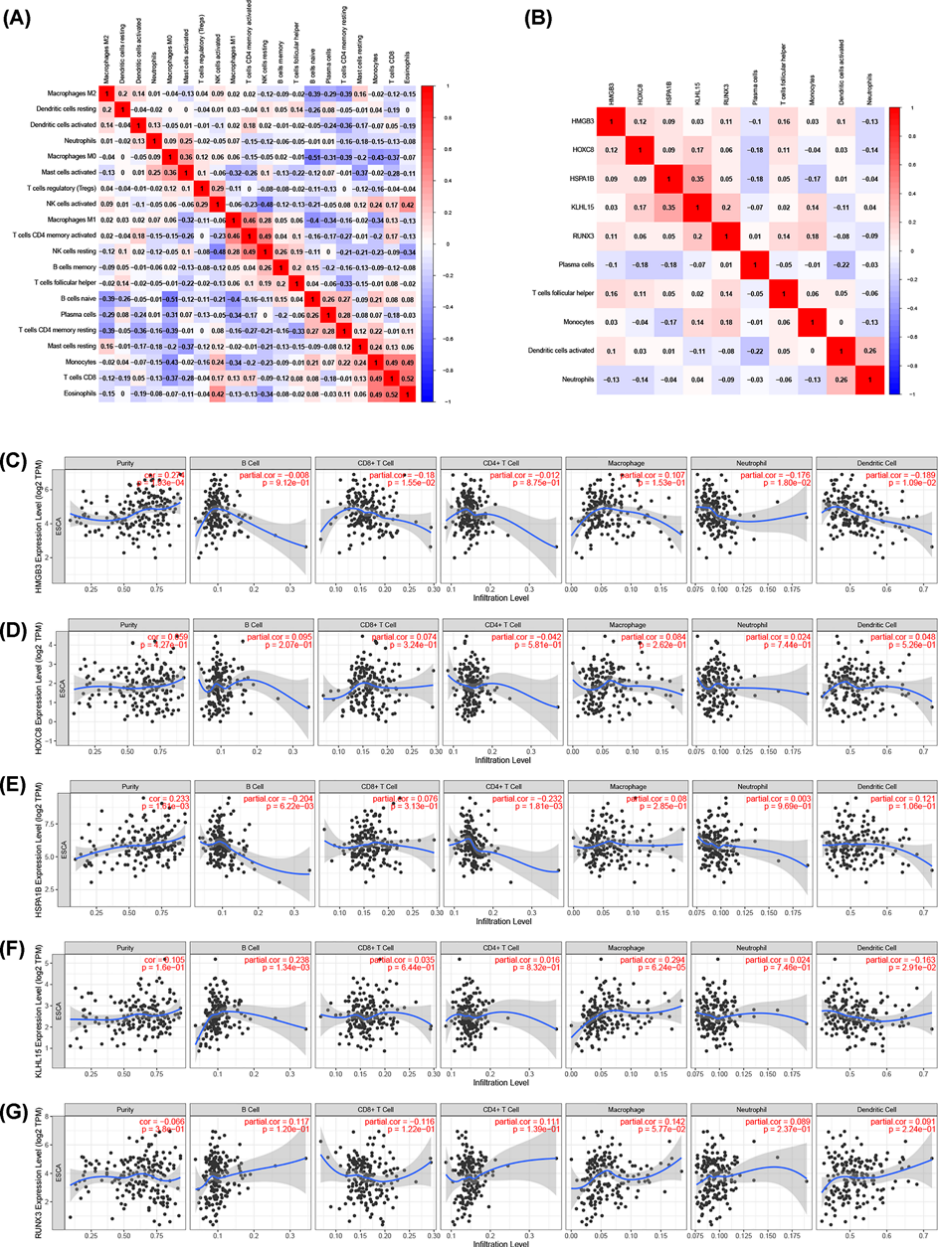
Correlation analysis of the key genes and immune cells (**A**) Correlation heat map of all immune infiltration cells. (**B**) Correlation heat map of key genes and immune cells involved in the prognostic models. (**C–G**) Correlation between key genes expression and six types of immune cells in the TIMER database.

We assessed possible correlations between 22 kinds of immune cells (Figure 6A), and Figure 6B showed the important co-expression patterns between the five immune cells and the five key molecules involved in the ceRNA network. The results showed that HMGB3 was positively correlated with follicular helper T cells (*R* = 0.16, *P*=0.049). HOXC8 was negatively associated with plasma cells (*R* = −0.18, *P*=0.024). RUNX3 was positively associated with monocytes (*R* = 0.18, *P*=0.021). HSPA1B was negatively associated with monocytes (*R* = −0.17, *P*=0.032) and plasma cells (*R* = −0.18, *P*=0.022) (Supplementary Figure S1). Besides, the correlations obtained from the TIMER database are generally consistent with the results above (Figure 6C–G).

## Discussion

ESCA is one of the most commonly diagnosed cancers in the world which is associated with poor prognosis and high mortality rate [21]. Thus, exploration of effective molecules and prediction models of ESCA could be helpful for its diagnosis and treatment.

In the present study, we focused on the ceRNA network and TME infiltrating immune cells. We identified 3211 DEmRNAs, 381 DElncRNAs, and 144 DEmiRNAs based on the TCGA database. A ceRNA network was constructed. Five key molecules were identified from the ceRNA network and five significant immune cells were identified via CIBERSORT. Two risk models were established on the base of the significant TME infiltrating immune cells and mRNAs. The high AUC values of both prognostic models indicated the advantage in predicting OS of ESCA patients. GO term and KEGG pathway enrichment analysis suggested that the DEmRNAs involved in the ceRNA network were mostly involved in cell cycle checkpoint and p53 signaling pathway. Previous studies revealed that dysregulation of cell cycle process played a vital role in the progression of tumors [22]. P53 signaling pathway is a typical tumor suppressor pathway, which is related to many types of cancer [23]. It has been reported that SASS6 can promote the proliferation of esophageal squamous carcinoma cells by inhibiting the p53 signaling pathway [24]. Among the ceRNA network, five significant molecules including HMGB3, HOXC8, HSPA1B, KLHL15, and RUNX3 could be considered as independent prognostic factors according to the results of multivariate Cox regression. HMGB3, high mobility group-box 3, is a member of the high mobility group box subfamily [25]. It has been proved that HMGB3 can affect the occurrence and development of many tumors. A recent study showed that HMGB3 was up-regulated in breast cancer, and silencing HMGB3 can inhibit breast cancer cell proliferation and tumor growth [26]. HMGB3 has also been reported as a potential prognostic marker in colorectal cancer. It can promote growth and migration of colorectal cancer via activating WNT/β-catenin pathway [27]. In addition, the high expression of HMGB3 is also an important prognostic indicator of low survival rate in patients with esophageal cancer [28]. HOXC8 is one of the HOX family genes. The HOX genes are significant factors in the regulation of embryogenesis. They encode a set of transcription factors and regulate the expression of downstream target genes through specific DNA binding [29]. In the past decade, HOX genes have been proved to be dysregulated in many solid tumors [30]. Furthermore, over-expression of HOX gene was related to poor prognosis [31–33]. HSPA1B is a member of the heat shock protein 70 (HSP70) family. It is reported that the expression of HSPs was higher in various tumors and closely associated with tumor progression [34]. HSPA1B could be considered as a promising target for prognostic prediction in esophageal cancer [35]. RUNX3, runt-related transcription factor 3, is a tumor suppressor gene involved in the TGF-β signaling pathway [36]. Previous studies have indicated that RUNX3 is a tumor suppressor gene for several human cancers including esophageal cancer [37]. The low expression level of RUNX3 showed worse prognosis for ESCA patients [38]. The protein encoded by KLHL15 may be involved in protein ubiquitination and cytoskeletal organization. Nevertheless, the specific role of KLHL15 in ESCA needs to be further explored.

Then, to explore the difference of tumor microenvironment between ESCA samples and normal samples, we observed the immune cell composition between normal and tumor groups. We found that Macrophages M0 and dendritic cells activated were obviously higher in ESCA samples, while CD8 T cells and CD4 T cells memory resting were obviously higher in normal samples. Tumor-associated macrophages, a major immune component of a variety of tumors, could promote tumor progression via secreting pro-angiogenic and growth factors [39,40]. Macrophages M0 can be polarized into different phenotypes, including macrophages M1 and macrophages M2 [41]. Some studies have indicated that macrophages M1 participated in pathogen clearance and proinflammatory response, while macrophages M2 were anti-inflammatory and correlated with tumor progression [42]. Furthermore, it is reported that high infiltration of tumor-associated macrophages is associated with worse prognosis of ESCA patients [43,44]. Dendritic cells are potent antigen-presenting cells, and the infiltration of dendritic cells in a tumor is considered the host’s immune response to the tumor [45]. They provide costimulatory molecules and cytokines, which provide necessary signals for the activation and differentiation of T cells, thus form the immune response [46]. In addition, they can activate anti-tumor responses via interacting with other immune cells, including NK cells and B cells [47,48].

T cells, including CD4 and CD8, were important components of TME and closely correlated with the development and prognosis of tumors [49]. Cytotoxic T lymphocytes (CTLs), which mainly express T cell co-receptor CD8, are closely associated with anti-tumor immune response [50]. It has been reported that CD8 T cells in esophageal cancer were related to positive OS [51]. Moreover, a recent study showed that high CD8 T cells and CD4 T cells infiltration have the potential to be prognostic markers and predict better OS in ESCA patients [52].

However, our study is limited because we need a larger sample size to validate our results, and the two prognostic models need more samples to further confirm its predictive ability. Furthermore, the present study is only a multi-dimensional correlation research, thus further experiments are necessary for better understanding the functions of the hub genes in ESCA.

## Conclusions

On the basis of tumor-infiltrating immune cells and the ceRNA network, two predictive models were constructed to predict the prognosis of ESCA patients. Five key molecules including HMGB3, HOXC8, HSPA1B, KLHL15, and RUNX3 could be considered as independent prognostic factors. In addition, the significant immune cells described in the study might play a vital role in the occurrence and development of ESCA. In conclusion, the present study provides an effective bioinformatics basis for exploring the molecular mechanism of esophageal cancer and predicting its prognosis.

## Supplementary Material

Supplementary Figure S1

Supplementary Tables S1-S5

## Data Availability

All data were downloaded from TCGA (https://portal.gdc.cancer.gov/).

## Abbreviations

ceRNA: competing endogenous RNA
DEG: differentially expressed gene
ESCA: esophageal cancer
LASSO: least absolute shrinkage and selection operator
OS: overall survival
ROC: receiver operating characteristic
TCGA: The Cancer Genome Atlas
