# Supplementary material for: Integrated analysis of ceRNA network and tumor-infiltrating immune cells in esophageal cancer
Source: Biosci Rep. 2021 May 27;41(5):BSR20203804. doi: 10.1042/BSR20203804 (PMC8164107; doi:10.1042/BSR20203804)
Supplement: Supplementary Figure S1 [file BSR-2020-3804_supp.pdf]

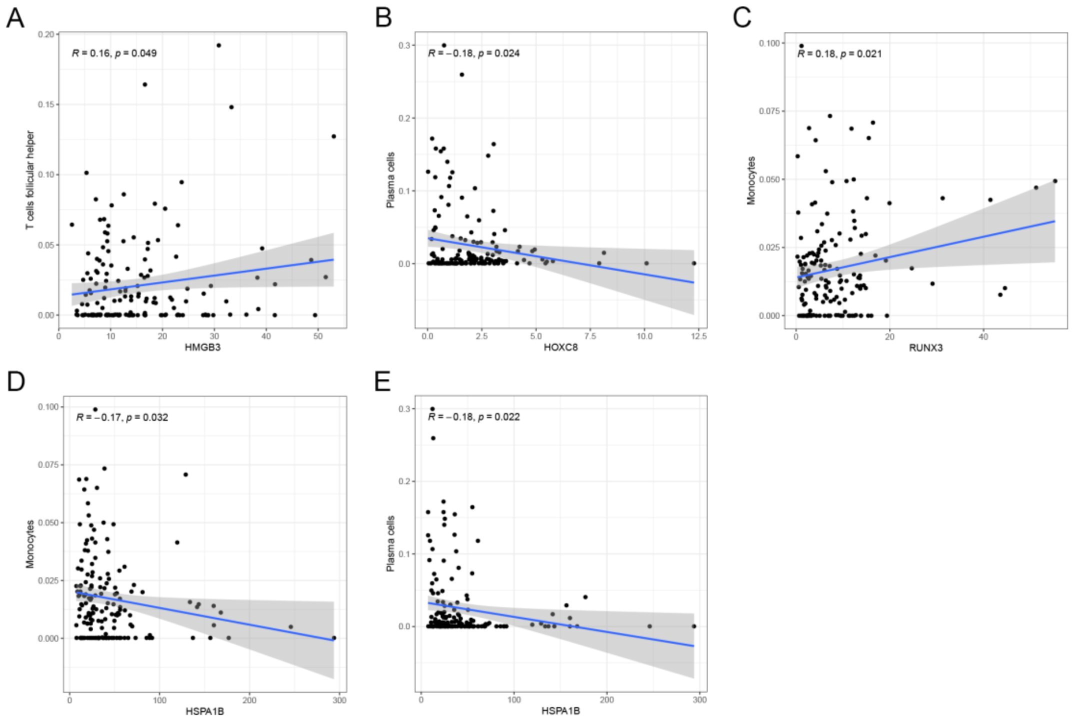

Figure S1: The significant co-expression patterns between the immune cells and the key molecules involved in the ceRNA network. (A) Correlation between HMGB3 expression and follicular helper T cells. (B) Correlation between HOXC8 expression and Plasma cells. (C) Correlation between RUNX3 expression and Monocytes. (D) Correlation between HSPA1B expression and Monocytes. (E) Correlation between HSPA1B expression and Plasma cells.
